# Neprilysin regulates the progression of glioblastoma: an in-vitro study using siRNA mediated gene silencing and HDAC1 mediated upregulation of neprilysin in U87 MG cells

**DOI:** 10.1007/s11010-025-05341-9

**Published:** 2025-06-29

**Authors:** Adarsh Gopinathan, Runali Sankhe, Raghavendra Upadhya, K. Sreedhara Ranganath Pai, Anoop Kishore

**Affiliations:** 1 Department of Pharmacology, Manipal College of Pharmaceutical Sciences, Manipal Academy of Higher Education, Manipal, Karnataka 576104 India; 2 Manipal Centre for Biotherapeutics Research, Manipal Academy of Higher Education, Manipal, Karnataka 576104 India

**Keywords:** Neprilysin, Glioblastoma, Histone deacetylase, Gene silencing, siRNA

## Abstract

Neprilysin (NEP) is a neutral endopeptidase that has gained attention due to its ability to cleave diverse peptides such as fibroblast growth factor-2 (FGF-2), insulin-like growth factors (IGFs), substance P, amyloid-β, thymopentin etc. NEP plays an important role in the functioning of the central nervous system, cardiovascular system, and in pathologies such as Alzheimer’s disease, hypertension, and various cancers. In breast, ovarian, prostate, and lung cancers, reduced NEP levels are associated with cancer progression. In Glioblastoma (GBM), the level of NEP is downregulated. This study aims to understand the role and expression pattern of NEP in GBM. A web-based tool, UALCAN, was utilized to understand the expression pattern of NEP in GBM, followed by patient survival analysis using the Cancer Genome Atlas (TCGA) data. Further, *in-vitro* scratch assays were performed on U87 MG cells to understand the effect of NEP silencing, as well as its upregulation using certain HDAC1 inhibitors identified through *in-silico* studies (melphalan, tasimelteon and panobinostat), to study the cancer progression. The UALCAN web tool revealed that NEP levels are downregulated in GBM. Additionally, the *in-vitro* scratch assay demonstrated that silencing of NEP augmented cell proliferation, whereas the upregulation of NEP using the HDAC1 inhibitors resulted in decreased cancer proliferation. These results suggest an inverse correlation between the NEP levels and GBM proliferation. The tumor suppression exhibited by NEP could be attributed to its degradation of mitogenic proteins such as FGF-2, IGFs etc. In conclusion, NEP can be a promising biomarker and a drug target against GBM.

## Introduction

Neprilysin (NEP), also known as CD10 (cluster of differentiation 10) or MME (Membrane metalloendopeptidase) [1], is a zinc-activated endopeptidase that cleaves peptides up to 50 amino acids long [2]. It is an integral membrane protein with its catalytic domain present on the extracellular C-terminal [3]. The substrates of NEP include fibroblast growth factor-2, insulin-like growth factors, substance P, bradykinin, atrial natriuretic peptide, angiotensins, endothelin 1 & 2, amyloid-β, endorphins, bombesin-like peptides, glucagon, thymopentin etc. [4]. The presence of NEP has been reported in tissues of the brain, kidney, heart, lungs, brain, placenta, thyroid, genital tract, gastrointestinal tract, etc. [5, 6]. Due to its ability to cleave various peptides, NEP has gained considerable attention in recent years. Several studies suggest that the NEP’s peptide-degrading actions makes it a key player in multiple pathological conditions [5].

NEP has an important role in cancer progression [6]. It regulates the levels of phosphatase and tensin homologue (PTEN), focal adhesion kinase (FAK), fibroblast growth factor-2 (FGF2), phosphatidyl inositol 3-kinase (PI3K)/Akt and neuropeptide signalling, which are crucial in the progression of cancer [7]. By controlling the levels of growth factors and oncogenic signalling molecules, NEP exerts a protective role in cancers of breast, lung, ovary etc. [6, 8]. It is reported that progression of cancer accelerated when NEP expression is downregulated [9].

The primary reasons for the downregulation of NEP include hypermethylation of the promoter region of NEP, and AICD (Amyloid Precursor Protein Intracellular Domain) mediated histone modification [10]. The AICD-mediated histone modification is an epigenetic modification responsible for the downregulation of NEP gene expression. Histone deacetylase (HDAC) enzymes, especially the HDAC1, compete with AICD for the promoter region of NEP gene [7], leading to suppression of NEP gene transcription. Furthermore, studies have shown that HDAC1 levels are upregulated in glioblastoma and silencing HDAC1 leads to an increase in P53 levels, thereby activating apoptosis [11, 12]. Additionally, P53 upregulates both p21^WAF1/CIP1^, a tumour suppressor gene, and Bax, a promoter of apoptosis. Studies in cancer cells have shown that HDAC1 represses both p21^WAF1/CIP1^ and Bax, contributing to uncontrolled cell proliferation [13]. Treatment with HDAC1 inhibitors cause release of HDAC1 protein from the Sp1 (Promoter-specific RNA Polymerase II transcription factor), and promote p21 transcription and regulation of tumor growth [14]. The HDAC1 levels are elevated in several cancers such as colorectal, lung, gastric, and bladder cancers. Gliomas also exhibit a significantly higher expression of HDAC1 [15]. This was further supported by a study conducted on glioma tissues, where a direct correlation was observed between HDAC1 levels and cancer progression [16]. Additionally, studies conducted on prostate cells treated with HDAC inhibitors like valproic acid and trichostatin A showed an increased in NEP gene expression and elevated NEP protein levels [17]. Therefore, HDAC1 inhibitors are potential candidates to upregulate the levels of NEP levels and control the progression of tumors.

Around 75% of the tumors occurring in the brain are gliomas, and glioblastoma multiforme (GBM) accounts for over 50% of all gliomas. According to World Health Organization (WHO) classification of tumors (WHO CNS 5), astrocytic tumors are divided into- with and without isocitrate dehydrogenase (IDH) mutations. Tumors without IDH mutations (wildtype) are termed as glioblastoma (IDH- wildtype with CNS WHO grade 4) [18]. GBM is one of the most aggressive and heterogeneous brain cancers [19] that emerges from astrocytes, which are a subtype of the non-neuronal cells that provide homeostatic support to the neurons in the nervous system [20, 21]. If untreated, GBM survival outcomes are bleak, with a median survival rate of 6 months [22]. It is one of the fastest growing tumors and among the deadliest form of cancers [23].

The standard treatment regimen for GBM includes tumour resection, radiotherapy, and temozolomide chemotherapy. However, the tumour recurrence in patients remains common [24, 25]. The complete resection of the tumour is difficult to achieve through chemotherapy and radiotherapy, since these treatments enhance the chances of toxicity to healthy tissues, potentially leading to neurological complications [26]. The current challenges in GBM treatment include incomplete tumour resection, tumour heterogeneity and impermeable blood–brain barrier (BBB) [27]. Several studies conducted on the human brain have identified the expression of NEP in astrocytes [28, 29]. Since GBM is an astrocytoma, [30] and the role of NEP has not been evaluated in glioma till date, we aimed to explore the effect of suppression as well as upregulation of NEP levels on the progression of GBM. We used siRNA to silence NEP gene to study its effect on GBM. Additionally, our previous finding on the identification of HDAC1 inhibitors through *in-silico* drug repurposing approach resulted in the selection of 3 most potent molecules- melphalan, tasimelteon and Panobinostat, whose effect on NEP upregulation was also evaluated. We report that the NEP levels are downregulated in the GBM, and the inhibition of HDAC1 elevated the levels of NEP protein in-vitro.

## Methodology

### Assessing NEP gene expression in glioblastoma

The expression of NEP gene in a few selected human cancers was assessed using the interactive web-portal- UALCAN (The University of ALabama at Birmingham CANcer data analysis Portal) [31]. UALCAN uses data obtained from *The Cancer Genome Atlas* (TCGA) to evaluate protein expressions in several cancers. To obtain the data on NEP expression, we performed the following keyword searches, Gene- MME, Cancer type- GBM and Analysis type- GBM vs Normal sample. Also, the expression of NEP in specific subsets within GBM, such as patient age groups and gender was also analysed. Further, the patient survival analysis and the NEP promoter methylation status was evaluated using MethMarkerDB, a database that utilizes the data from TCGA, which consists of 450 K methylation array data, RNA-Seq data and clinical data to construct prognostic and diagnostic models [32].

### Cell culture and maintenance

U87MG (human glioblastoma) cells were purchased from NCCS (National Centre for Cell Science), Pune, India. The cells were grown in a T75 cm^2^ flask in DMEM media (Dulbecco’s Modified Eagle Medium from Gibco, Thermo Fisher Scientific) containing 10% FBS (Fetal Bovine Serum from Gibco, Thermo Fisher Scientific), 100 units/ml penicillin, 50 μg/ml streptomycin and 100 μg/ml amphotericin (Invitrogen, Thermo Fisher Scientific) at 37 °C, in a 5% carbon dioxide maintained atmosphere (CO_2_ incubator, New Brunswick Galaxy 170 S, Eppendorf, USA).

### Neprilysin silencing and scratch assay

To study the effect of NEP silencing on the growth of GBM *in-vitro*, in cultured U87MG cells, the NEP gene was silenced using an siRNA, and the scratch assay was performed. For NEP silencing, siRNA specific for human NEP/CD10 was procured from Santa Cruz Biotechnology, Inc., TX, USA (Cat No. 29959). The reagents used for transfection includes Lipofectamine 2000 (Cat No. 11668019, Invitrogen, Thermo Fisher Scientific) and Opti-MEM, reduced serum media (Cat No. 31985070, Gibco, Thermo Fisher Scientific), gene silencing performed according to the manufacturer’s instructions and according to the validated protocol [33]. U87 MG cells were transfected with different concentrations (20,40 and 60 picomolar) of NEP siRNA and western blot was performed at 48 h and 72 h to quantify NEP silencing. Then, scratch assay was performed [34] after transfecting the cells with NEP siRNA using the concentration and time at which maximum NEP gene silencing was observed.

### Effect of neprilysin on growth of GBM cells *in-vitro*

To determine the effect of external NEP, Biolegend (Cat. No. 790502), administration on the growth of U87 MG cells, various concentrations of NEP, such as 22.5, 225 and 450 picomols of the protein were added and the scratch assay was performed.

### Scratch assay

The *in-vitro* scratch assay is a standard technique to identify cell migration. In this technique, a scratch is created in a confluent monolayer of cells with the help of 1 ml pipette tip. Then cells are given PBS wash to remove any cell debris followed by treatment. Finally, the migration of cells was observed under microscope at 0, 24 and 48 h [35]. In the scratch assay, the percentage area closure was determined using the formula [34]-.

$$\% {\text{ Area closure}} = \, \left[ {{\text{A }}\left( 0 \right) - {\text{ A}}\left( {\text{t}} \right)/{\text{A }}\left( 0 \right)} \right] \, \times {1}00$$

Here A (0) represents the area at time (0) and A(t) represents the area at time (t).

### Effect of *in-silico* repurposed HDAC1 inhibitors on NEP levels and activity in U87 MG cells

Downregulation of NEP is implicated in the growth progression of several cancers. One of the major reasons for downregulation of NEP is the action of histone deacetylases on NEP gene, especially the HDAC1. In our previous work, based on *in-silico* drug repurposing approach [36], we had screened 1379 FDA-approved drugs from the ZINC15 database to identify the three most potent HDAC1 inhibitors– Panobinostat, Melphalan and Tasimelteon. *In-vitro* studies using rat (C6 cells) and human (U87MG) glioblastoma cells revealed that, treatment with all the three compounds significantly lowered the levels of elevated HDAC1 in the cultured cells. Therefore, in the current study, we also evaluated whether these molecules could elevate NEP levels in GBM, due to their ability in reducing HDAC1. For this, U87 MG cells were treated individually with the IC_10_ and IC_30_ doses of these drugs. After 48 h of incubation, the cells were harvested, and cell lysates were prepared for western blot analysis and NEP activity assay. The NEP activity was estimated fluorometrically using a commercially available assay kit (MAK350, Sigma-Aldrich Co. LLC, St. Louis, MO, USA). Additionally, scratch assay was performed after treating the cells with the IC_10_ and IC_30_ doses of these drugs. In the study, the therapeutically used drug for glioblastoma- Temozolomide, was also included as a treatment group for comparison of the efficacy of the compounds.

### Western blotting

The treated U87MG cells were collected, and cell lysates were prepared. In the cell lysates, the total protein content was estimated using the bicinchoninic acid assay (BCA protein assay kit, Thermo Fisher Scientific Inc.). Lysates equivalent to 30 µg of protein were resolved in 10% Tris–HCl SDS PAGE (Sodium dodecyl sulphate- Polyacrylamide gel). The gels were transferred to PVDF membrane. Then, the membranes were incubated for blocking in 5% bovine serum albumin (BSA) dissolved in Tris buffered saline (TBS) containing 0.1% Tween 20, for 2 h at room temperature. Subsequently, incubated the blots overnight at 4 °C, with 1:500 dilution of NEP antibody (Elabsciences, E-AB-12794) and 1:1000 dilution of α-tubulin (Elabsciences, E-AB-20069) used as housekeeping standard. Then, the membranes were washed and incubated with the secondary antibody having 1:10,000 dilution (anti-rabbit IgG conjugated with horseradish peroxidase). The bands were visualized using a chemiluminescent HRP substrate (Takara Bio Inc., Shiga, Japan). Densities of the protein bands were analysed using the software ImageJ (version 1.54j, NIH, Bethesda, MD, USA) [37].

### Statistical analysis

The values are expressed as Mean ± SEM. The NEP levels are expressed as the relative band density (i.e., ratios of band intensities of NEP to that of the α-tubulin band). The statistical analyses were performed using Prism (version 8.4, GraphPad Software Inc., La Jolla, CA, USA). *p* < 0.05 was considered statistically significant. All the experiments were performed in triplicates. One-way ANOVA followed by Tukey’s test was performed for western blot and NEP activity. Two-way ANOVA followed by Tukey’s test was performed for scratch assay.

## Results

### The expression NEP in cancers

**Fig. 1:**
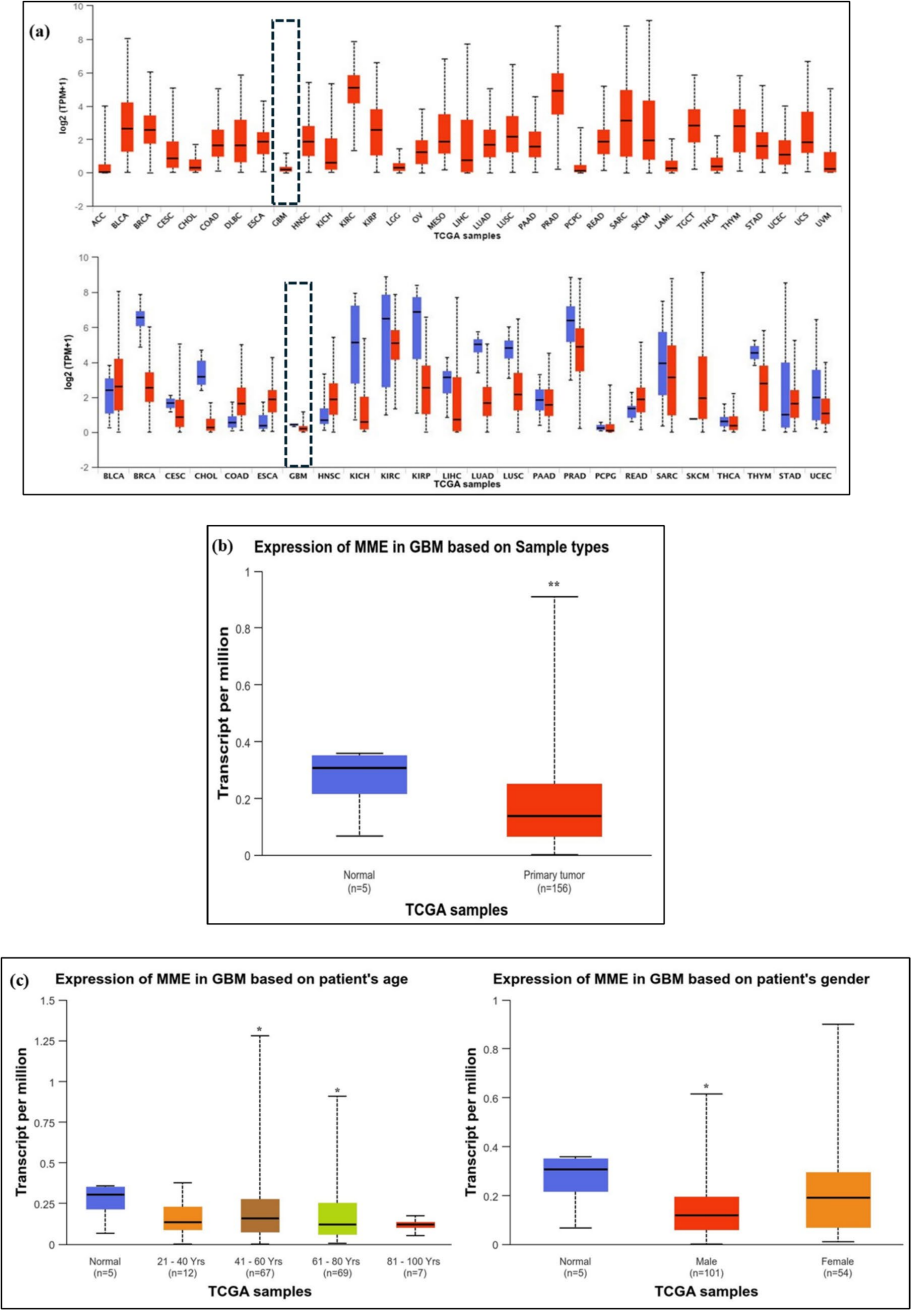

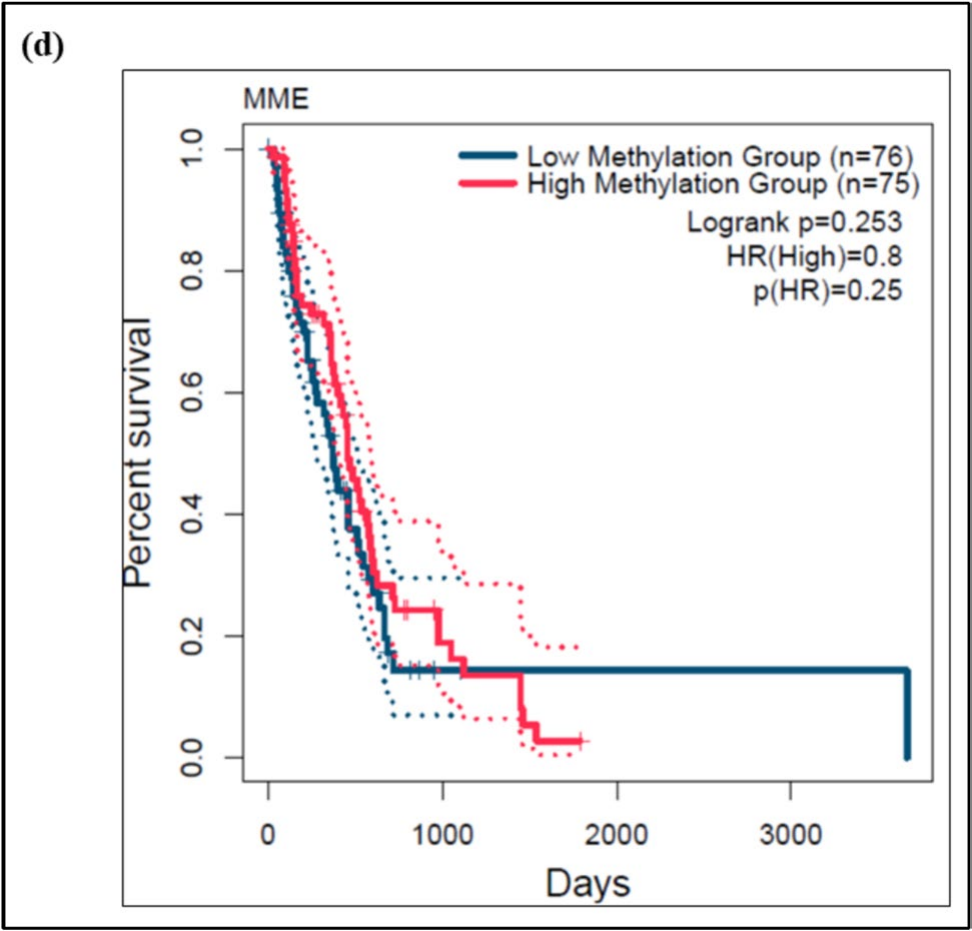
The figure depicts the **a** Pan cancer view of NEP/MME expression in tumour and normal samples **b** Expression of NEP/MME in normal and tumour samples of GBM, **c** Expression of NEP/MME in GBM based on Patient’s age and gender and **d** Kaplan–Meier plot of GBM patients having promoter region methylated along with survival analysis, where *p < 0.05 and **p < 0.01 compared to the normal sample

The expression of NEP/MME across TCGA samples is shown in Fig. 1. The findings indicated that the NEP levels are lower in GBM across all the TCGA tumour samples (Fig. 1a). The expression of NEP in tumour samples was significantly less than in the normal samples at p = 0.0029 (Fig. 1b). Similarly, the expression level based on age in GBM revealed that the age group between 41 and 60 years (p = 0.0118) and between 61 and 80 years (p = 0.0287) showed statistically significant reduction in the NEP levels than the normal sample. However, the other age group in GBM did not show any statistical difference in the NEP levels than the normal samples. Additionally, the expression of NEP in gender (male and female) in GBM was also evaluated (Fig. 1c). The results revealed lower expression of NEP in males (p = 0.0154) than the females compared to the normal samples GBM. The effect of promoter methylation and survival of GBM patients was analyzed using the Kaplan–Meier plot. There was no significant (p = 0.253) difference between the low and high methylation groups (Fig. 1d).

### Effect of NEP silencing

**Fig. 2:**
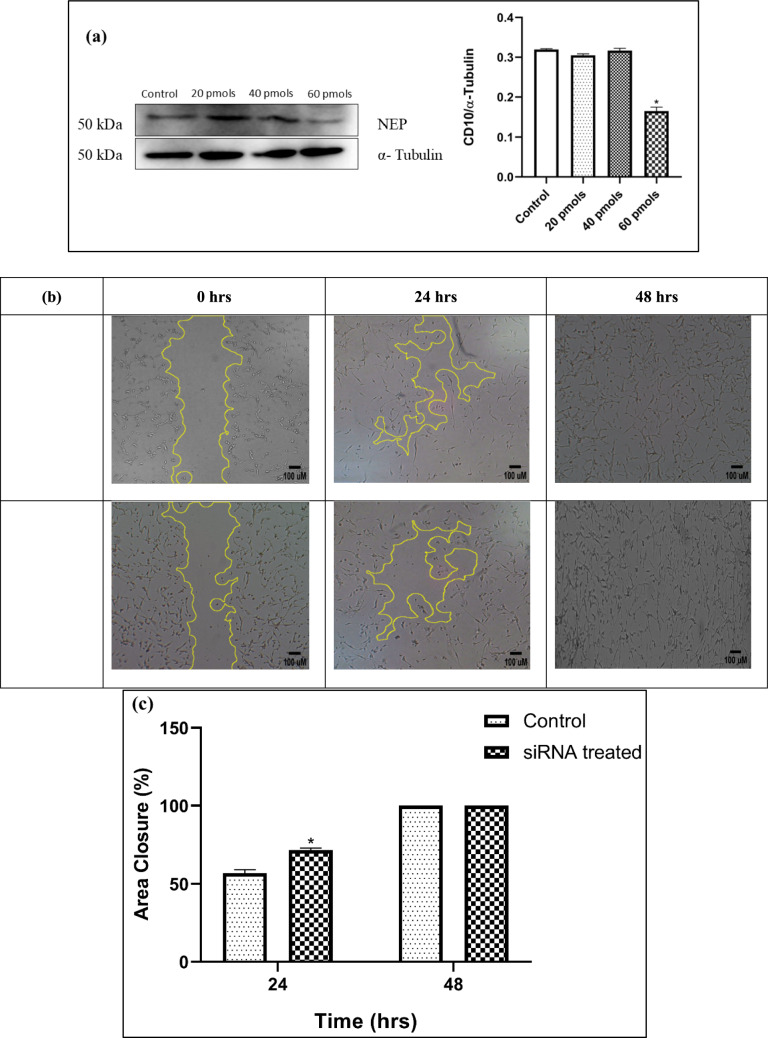
**a** A representative image of western blot analysis of NEP silencing after 48 h in U87 MG cells using 20 pmols, 40 pmols and 60 pmols of NEP siRNA and relative density ratio of NEP/α-tubulin. **b** The scratch assay on U87 MG cells was captured on 0, 24 and 48 h. **c** The graph depicts the Percentage Area closure (%) vs Time (hrs) between control and siRNA-treated samples in U87 MG cells. The values are represented as mean ± SEM, where *p < 0.05 vs the control group (n = 3)

The optimum amount of siRNA to silence NEP protein was determined by treating the cells with various concentrations of NEP siRNA. Treatment for 48 h with 60 pmols of siRNA specific to NEP resulted in decreased levels of the protein, which was confirmed by lesser band intensity (at 50 kDa) by western blot analysis (Fig. 2a). The same concentration of siRNA was used to perform the scratch assay and no change in area closure was observed in the treatment group compared to the control group for 48 h (Fig. 2b and c). The areas under 24 and 48 h were utilized for calculation.

**Fig. 3:**
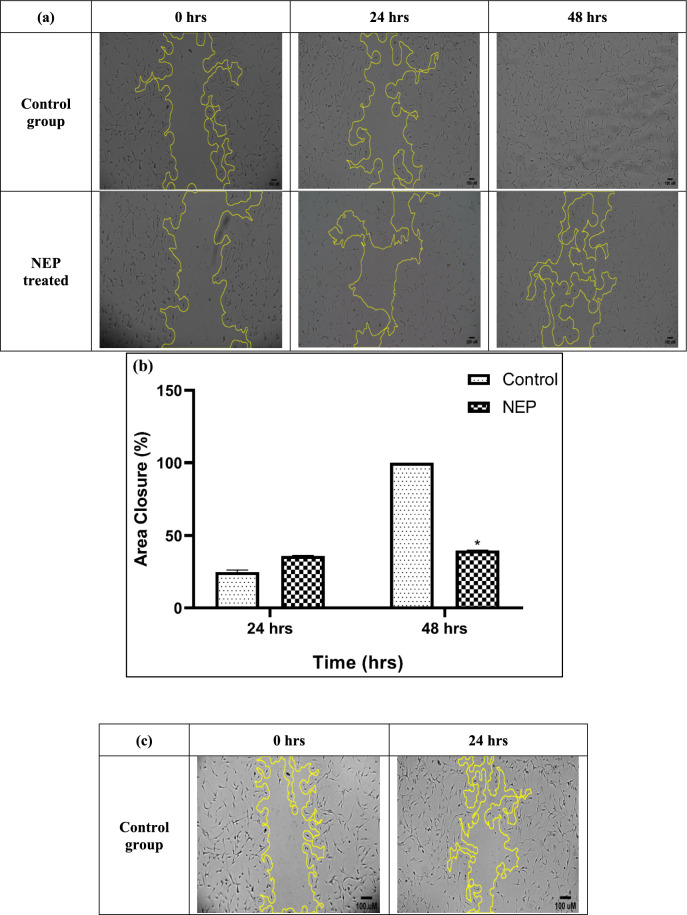

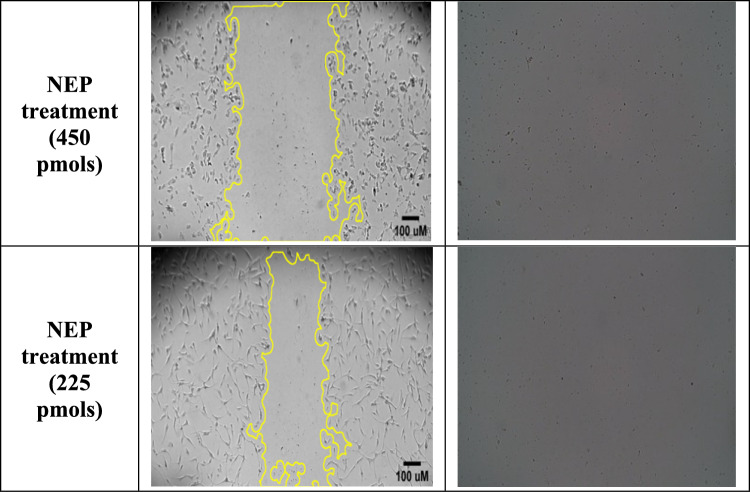
**a** The image of the scratch assay on U87 MG cells using 22.5 pmols captured on 0, 24 and 48 h. **b** The graph depicts the Percentage Area closure (%) vs Time (hrs) between control and NEP treated samples in U87 MG cells, *p < 0.05 vs control group. **c** The scratch assay on U87 MG cells using 450 pmols and 225 pmols captured on 0 and 24 h. At 24 h, both the treatment groups showed cell death compared to the control group

Next, the cells were incubated with various concentrations of NEP protein (22.5, 225 and 450 pmols). At 48 h, a significant reduction (at p < 0.05) in the area of closure was observed in the cells treated with 22.5 picomolar solution of NEP, compared to untreated cells (Fig. 3a). However, incubation with 225 and 450 pmols of NEP resulted in cell death by 24 h (Fig. 3b and c). These results indicate that NEP might have a significant regulatory role in cancer migration.

### Effect of *in-silico* repurposed HDAC1 inhibitors on NEP levels, activity and wound closure in U87 MG cells

**Fig. 4:**
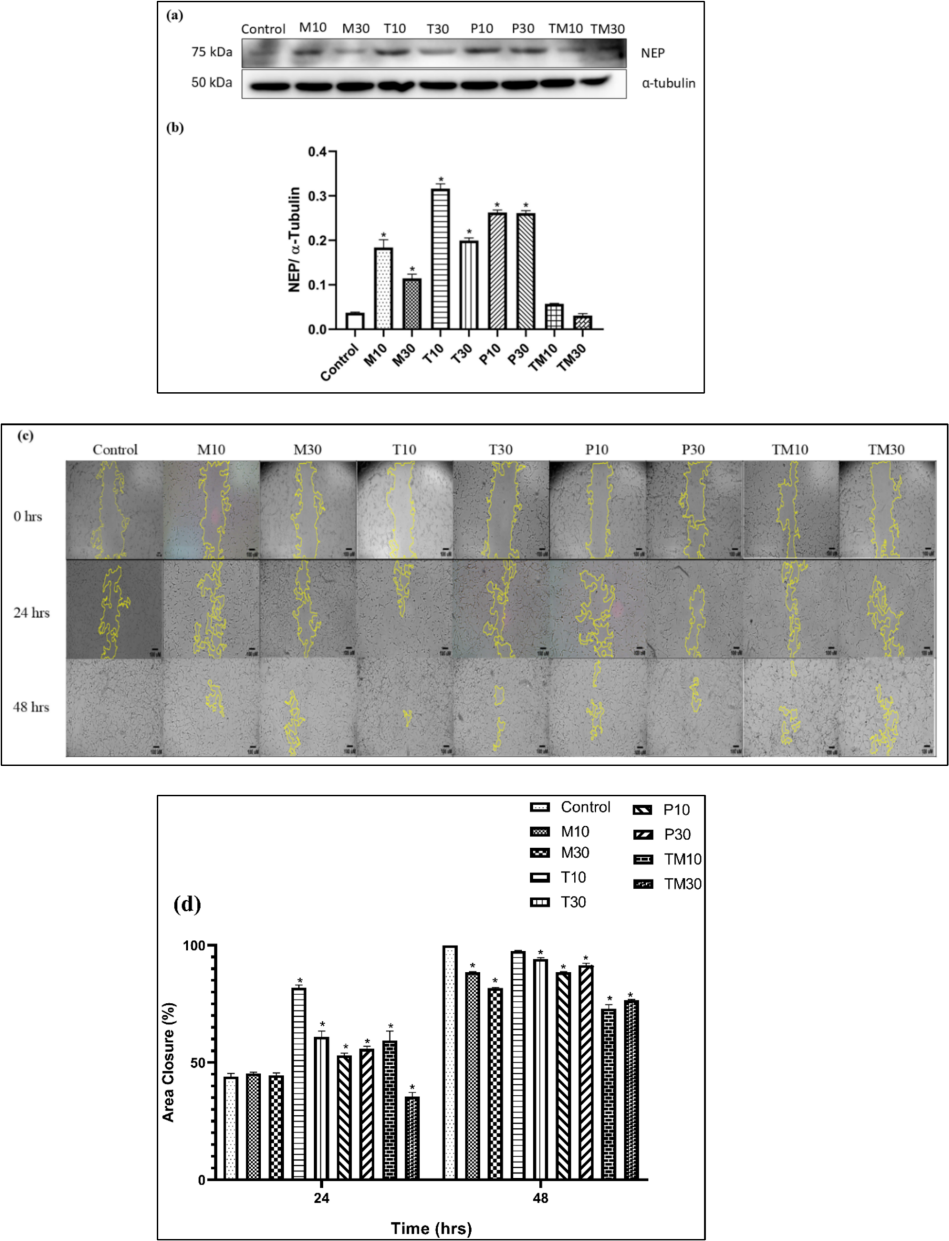
A representative image of **a** western blot analysis of NEP protein in U87 MG cells and **b** relative density ratio of NEP/α-tubulin. The values are represented as mean ± SEM, where *p < 0.05 vs control group (n = 3). **c** Scratch assay on U87 MG cells after drug treatment captured on 0, 24 and 48 h. **d** The graph depicts the Percentage Area closure (%) vs Time (hrs) between control and drug treated samples in U87 MG cells, *p < 0.05 vs control. 10 and 30 represent the IC_10_ and IC_30_ doses of *M* Melphalan, *T* Tasimelteon, *P* Panobinostat and *TM* Temozolomide respectively

U87 MG cells were treated with the IC_10_ and IC_30_ doses of panobinostat (0.018 nM and 3.63 nM), melphalan (54.30 μM and 213.91 μM), tasimelteon (195.97 μM and 559.86 μM) and temozolomide (288.85 μM and 957.93 μM). The results showed that the IC_10_ and IC_30_ doses of melphalan, tasimelteon and panobinostat showed a significant increase in the NEP levels at p < 0.05, compared to the untreated control group. However, in the temozolomide treatment group no significant increase in the NEP levels was observed (Fig. 4a and b).

The effect of these molecules on cell migration was performed and all the molecules except tasimelteon (IC_10_) showed a significant decrease in the area closure compared to the untreated control group after 48 h of incubation (Fig. 4c and d).

**Fig. 5:**
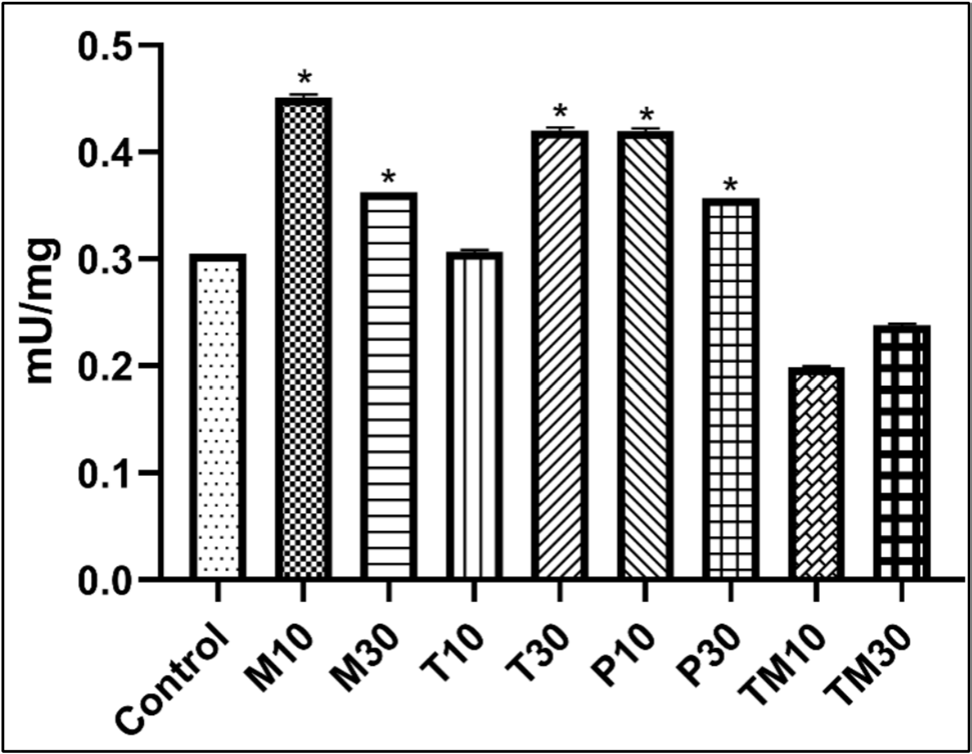
The effect of treatment groups on NEP activity in U87 MG cells. Where *p < 0.05 with respect to control. Values are expressed as mean ± SEM.10 and 30 are the IC_10_ and IC_30_ doses of *M* Melphalan, *T* Tasimelteon, *P* Panobinostat and *TMZ* Temozolomide

Followed by western blot analysis, the effect of drug treatment in U87 MG cells was analysed for NEP activity. After the drug treatment, the cells were harvested and treated with the assay buffer provided with the kit. Using a fluorescence microplate reader, the fluorescence was measured and calculated as per the manufacturer’s protocol. The drugs like melphalan (IC_10_ and IC_30_), tasimelteon (IC_30_) and panobinostat (IC_10_ and IC_30_) showed a significant increase in the NEP level compared to the untreated control group (Fig. 5). However, the IC_10_ dose of tasimelteon and both the doses of temozolomide failed to show any upregulation in the NEP activity compared to the untreated control group. The results obtained were similar to that of western blot analysis, where the standard drug failed to upregulate the NEP activity.

## Discussion

Several studies have reported an inverse correlation between NEP levels and tumour progression. A few of them, however, report contradictory results, with certain tumors overexpressing NEP [38]. Our study is the first in the glioblastoma, where we hypothesized that NEP levels would be downregulated in GBM.

Initially, we employed the UALCAN tool, a free web-based platform, across the TCGA dataset and confirmed that NEP levels are downregulated in GBM patients compared to the normal group, which shows that NEP levels inversely correlate with GBM progression. Suppression of NEP activity promotes the development and growth GBM, and increasing the levels of NEP in the system halts the progression of cancer. Therefore, NEP downregulation, either through hypermethylation of the promoter region or and AICD mediated histone modification, could play a crucial role in GBM.

Studies on Alzheimer’s disease (AD) have revealed that the NEP promoter region is rich in CpG islands. The dense CG regions are the main target of the DNA methyltransferase enzyme, enhancing methylation at the promoter region [39], which was supported by a study on prostate cancer that identified hypermethylation at the 5’ CpG island, resulting in the downregulation of NEP protein [40]. In our study, we tried to correlate NEP promoter region methylation with patient survival rate. However, there was no significant correlation between methylation levels and overall survival rate. This suggests that further studies need to be carried out to explore the role of methylation in NEP expression and its relationship with patient survival. Several reports have identified a circulating form of NEP (cNEP), a heterogeneous blend of moieties released in circulation, urine and cerebrospinal fluid (CSF) [41] due to proteolytic processes, giving fragments at 33 and 57–60 kDa [38, 42]. The bands obtained in our study were at 50 kDa and 75 kDa, indicating the presence of cNEP. The circulating form of the enzyme, cNEP, also has the potential to cleave the substrates throughout the body, including Amyloid β [43].

NEP is known to degrade several biologically active peptides such as fibroblast growth factor-2 and insulin-like growth factors that play an important role in tumour growth. Therefore, silencing NEP may have led to the accumulation of these growth-promoting factors, potentially contributing to tumour progression, in the in-vitro model of GBM. These results were supported by a study conducted on colon cancer, which showed that NEP is involved in colon cancer proliferation, growth and migration [44]. However, in contrast to NEP silencing, the directly treating the cancer cells with NEP showed reduced cell proliferation, possibly due degradation of pro-oncogenic peptides by the catalytic activity of NEP [6]. This observation was aligned with the study conducted on prostate cancer cells, where the addition of exogenous NEP resulted in reduced cell growth, migration, and tumorigenicity [45]. Additionally, treatment with 225 and 450 picomolar concentrations of NEP resulted in cell death at 24 h of incubation, which might be due to NEP toxicity to cells. Henceforth, further studies on exploring the action of NEP on various cellular processes are still required.

Like all other cancers, epigenetics plays a vital role in GBM progression. It has been reported that HDAC1 potentiates GBM cell proliferation by activating various signalling pathways, and HDAC1 has been linked to tumorigenicity in GBM stem cells [11]. So, inhibiting HDAC1 could upregulate NEP expression. Therefore, we selected the previously identified HDAC1 inhibitors like melphalan, tasimelteon and panobinostat, along with the standard drug, temozolomide to check NEP levels in the U87 MG cells. Panobinostat is a pan HDAC inhibitor that acts by inhibiting HDACs and it also reduces VEGF secretion, inhibiting angiogenesis in cancer. This molecule is in the phase II of clinical trial against GBM [46]. Tasimelteon, is a melatonin receptor agonist and no studies have been carried out using tasimelteon against glioblastoma. Previous studies have shown the direct relationship of melatonin with AICD. Studies conducted on mice models of Alzheimer’s have proven that melatonin causes elevation in AICD levels by the action of α-secretase and γ-secretase on APP, eventually increasing NEP levels [47]. Hence, this could be the possible mechanism through which tasimelteon treatment upregulated the NEP levels. Additionally, studies of melatonin on glioblastoma cells (U251 MG and U87 MG) have shown that melatonin exhibits anti-migratory action in a dose-dependent manner. The higher dose of melatonin (1 mM) has shown anti-migratory effects than a low dose (1 nM) by inhibiting the hypoxia-inducible factor (HIF-1α). Hence, that could be the reason in our study, the IC10 dose did not show the anti-migratory effect [48]. The other drugs, like melphalan and temozolomide, belong to the class of alkylating agents. The alkylating agents have already shown synergistic activity with HDAC inhibitors in HBL-2 cells [49]. Studies have shown that melphalan treatment can lead to the loosening of the chromatin structure, which might reduce the HDAC activity and also increase cell cycle arrest through P53 activation [50]. As HDAC1 inhibition is directly correlated to NEP expression, the HDAC inhibition followed by cell cycle arrest might be the possible reason for NEP upregulation.

This study is the first of a kind that tries to explore the relationship of these drugs with NEP levels through HDAC1 inhibition. Reports of the studies conducted on mouse models of Alzheimer’s have shown that HDAC1 inhibition elevated NEP levels [10]. Also, evidence supports the elevated levels of HDAC1 in GBM [12, 51], indicating a crosstalk between HDAC1 and NEP in GBM pathogenesis. Interestingly, in our results, melphalan (IC_10_ and IC_30_), tasimelteon (IC_10_ and IC_30_) and panobinostat (IC_10_ and IC_30_) showed an increase in NEP levels compared to the untreated control group. However, the standard drug, temozolomide failed to improve the NEP levels than the untreated control group. The results of NEP activity were consistent with the western blot results. This suggests that HDAC1 play a role in downregulating NEP levels, which can be reversed with HDAC1 inhibition. The NEP activity works on the principle of active NEP that cleaves the synthetic NEP substrate, O-amino benzoic acid (Abz) peptide to release fluorophore which is further quantified. Since the substrate is specific for NEP, it will provide a direct relationship between the generated fluorophore and NEP. Hence, the results of the western blot and activity assay indicate that the drug treatments have upregulated the NEP levels, except temozolomide. The effect of temozolomide on HDAC1 inhibition in U87 MG cells has been already studied [36], however further studies are required to understand the role of temozolomide in NEP regulation in U87 MG cells.

The U87 MG cells used in this study have limitations like the absence of tumour microenvironment, which is critical in GBM progression. The 2D monolayer of U87 MG cells lacks the cell–cell interaction, vasculatures, morphology, gene expression profiles etc. which are essential features of GBM and can be attained with a 3D model [52]. Similarly, tumour heterogeneity is one of the key concerns in GBM models, like the U87 MG cells exhibit the presence of wild-type P53 gene. However, around 40% of GBM has mutated or non-functional P53 status [53]. Furthermore, in this context, the role of other tumour-specific biomarkers needs to be evaluated to identify the potential relationship between HDAC1 and NEP.

## Conclusion

GBM is still one of the most aggressive and heterogeneous brain tumors. Several studies have reported alterations in NEP levels in many cancer models, and this is the first study to establish a relationship between reduced NEP levels and GBM. NEP generally acts by cleaving the peptide bonds on hydrophobic amino acids. Reduced NEP levels cause the accumulation of higher concentrations of biologically active peptides, leading to cancer cell proliferation. One of the significant causes of NEP downregulation is overexpression of HDAC1, and its inhibition through the repurposed drugs has shown an elevation in NEP levels in the *in-vitro* model. Together, our observations suggest that NEP can be a potential biomarker against GBM, provided further investigations on the role of NEP over various proliferative markers are required both in *in-vitro* and *in-vivo* model of GBM. Additionally, RNAseq analysis needs to be performed to understand the relationship of other genes with NEP.

## Data Availability

No datasets were generated or analysed during the current study.
